# From family estrangement to empowered exits: new emotional developments

**DOI:** 10.3389/fsoc.2025.1663618

**Published:** 2025-09-24

**Authors:** Andreea Nica

**Affiliations:** Department of Social Sciences and Cultural Studies, Western New Mexico University, Silver City, NM, United States

**Keywords:** emotion, estrangement, exit, resocialization, wellbeing

## Abstract

Adult child and parent estrangement has seen an upward trend in recent years. While former research has explored the motivations and implications of the family estrangement or exiting phenomenon, little is known about the emotional (re)socialization process of “exiters” in the family exiting process. Through in-depth, online interviews (*n* = 30), this qualitative study investigates how adult children and parent exiters undergo an emotional (re)socialization process related to wellbeing outcomes. The empirical findings reveal that the family exiting process is emotionally nuanced with social gains and consequences for both adult children and parents in this study. While respondents self-report positive feelings associated with severing ties, there is a mixed impact on overall wellbeing. This research has significant policy and practical implications in forging social and emotional support resources for individuals who experience an exit from their family system.

## Introduction

The emotion-based experiences of family estrangement have infiltrated the media (Coleman and Johnson, 2024; Frankel, 2025), legal domain, and autobiographies. Family estrangement is seeing an upward trend, and its prevalence in Western society has been documented (Collins, 2022; Conti, 2015; Pillemer, 2020). Previous studies have analyzed the hegemonic, ideological perspectives of the family system as significant and valuable (Agllias, 2011), in which biological family connections are expected to be permanent, close, supportive, and unconditional (Scharp and McLaren, 2017). The family system leverages capital—social, cultural, economic, emotional—that maintain its function in the social order (Costa et al., 2019). Thus, significant social consequences incur for those who exit their family system to improve their quality of life (Agllias, 2011; Scharp and Thomas, 2016).

Agllias (2011) defines *family estrangement* as “the physical distancing and loss of affection between family members, often due to intense conflict or ongoing disagreement” with indicators such as “lack of emotional intimacy, an unsatisfactory relationship, and a belief that there is no way to resolve problems” (p. 108). Regarding emotional intimacy, while perspective-taking, closeness, open communication, trust, and boundary management have been explored (Rittenour et al., 2018), studies have not yet examined *how* exiters (specifically adult child–parent exiters) undergo *emotional (re)socialization* related to wellbeing, and how *early family emotional socialization* informs resocialization.

This qualitative study extends *theoretical dimensions* of—emotional capital, work and management, and deviance—to conceptualize an emotional (re)socialization process related to wellbeing in family exits. From a sociological perspective, using the term “exiting” rather than “estrangement” in the context of family disconnection is important because “exiting” more accurately captures the agency, process, and structural embeddedness of leaving a family system, while “estrangement” tends to pathologize the phenomenon and obscure its social dimensions. It can individualize the experience by focusing on interpersonal conflict or psychological distress, and may overlook broader social, cultural, and institutional forces that shape family dynamics.

*Research Questions*: (1) What is the process of emotional (re)socialization for adult child–parent exiters? (2) How does adult child–parent exiters’ emotional (re)socialization inform their perceived wellbeing?

## Current literature

Motivations for estrangement include conflict, betrayal, abuse, addiction, illegal activity, poor parenting, as well as limited communication, disparate values, boundary conflict, and lack of emotional intimacy (Agllias, 2015; Blake et al., 2017; Gilligan et al., 2015). There are differences in causes and experiences of estrangement among adult children and parents (Carr et al., 2015). Parents report external stressors like family issues (e.g., divorce) or situational problems as causes, whereas children are inclined to report parental internal characteristics (e.g., perceived disrespect of values or boundaries, or lack of parenting skills) (Carr et al., 2015). Further, when families are more rigid in their beliefs, estrangement is likely to result from challenges to political, moral, and religious ideals (Davis, 2003; Sucov, 2006).

Some estrangement studies show that exiters experience negative emotions, depression, decreased ability to self-regulate, and heightened physiological response patterns (Friesen, 2003). Research also points to positive outcomes, which include greater independence, autonomy, and personal agency (Agllias, 2015, 2017). Exiters can also experience self-determination to pursue growth-oriented endeavors such as boundary management, self-development, and forging healthier relationships (Agllias, 2017; Linden and Sillence, 2021). Stigma is common, and exiters often feel the need to conceal their exit (Barnwell, 2024; Blake et al., 2020). Research on coping and therapies for exiters found some therapy approaches useful (e.g., validation), while other approaches were not (e.g., being pressured to reconcile or forgive) (Blake et al., 2020; Scharp, 2017).

## Theoretical framework

### Emotional (re)socialization

Sociologists are motivated to understand the conditions under which parental emotional socialization contributes to the development and distribution of emotional competence (or capital) and wellbeing (Erickson and Cottingham, 2014). In primary emotional socialization, individuals learn emotional expression and management in ways that reflect power-status relations (Kemper, 1978). This, in turn, prompts inquiry into how social control (in the family) transforms to personal agency and links to the maintenance or disruption of emotion normative order (Erickson and Cottingham, 2014). Research has not yet established an *emotional resocialization* process in family exits. By examining emotional resocialization, it advances theory by showing how family transitions and exits are not only relational or structural changes but also involve the dismantling and rebuilding of emotion norms that define family roles and hierarchy.

It further extends work on emotion norms by revealing how exiting families may require individuals to actively unlearn restrictive feeling rules (Hochschild, 2012) and potentially replace them with new emotional capital resources. It refines existing theories by showing that norms are not simply broken or resisted but reconstituted through resocialization, and that social identities in family transitions are anchored in new emotion norms. This positions family exiting as a critical site for observing how emotion norms, capital, management and work, and deviance interact and interrelate to wellbeing outcomes.

### Emotional capital

Empirical work expanding on Bourdieu’s conception of capital has framed *emotional capital* as cultivated in *primary socialization* and accumulated and compounded in *secondary socialization* (Bourdieu, 1986; Cottingham, 2016; Reay, 2000). Earlier research extends emotional capital to encompass a set of resources linked to affective family ties (Allatt, 1993; Nowotny, 1981). Bourdieu’s framework has also been applied to examining family-estranged students as disadvantaged when entering the higher education system (Bland, 2018; Costa et al., 2019). Cottingham (2016) uses emotional capital to refer to emotion-based knowledge, emotion management skills, and feeling capacities, as situated in power maintenance. Research reveals a subset of emotion intimacy skills, underpinning emotional capital, are practiced in emotional subcultures (secondary socialization) and enhanced wellbeing (Nica, 2022). Studies have not explored how emotional capital functions in emotional (re)socialization of family exits.

### Emotional management and work

*Emotional management* is an act of emotion regulation enacted in private life to cope with stressors (Hochschild, 2012; Lively and Weed, 2014). *Emotional work* is the management of feeling(s) to express an acceptable display in order to uphold emotion norms and social order; in addition, the emotion work required to manage private emotions (Hochschild, 2012). The use of emotional work and management skills depend on social status and are used to conform to *emotion norms* linked to structural arrangements but can lead to stress and inauthenticity (Thoits, 2004). In situating these concepts in the emotional (re)socialization of family exits, we can be informed on how exiters use emotional management skills and emotion work to navigate the tension of emotion norms conformity and deviance.

### Emotional deviance

Families are the primary institution in which emotional selves and norms are created and maintained (Erickson and Cottingham, 2014). When emotions deviate in quality or degree from what is expected in particular roles embedded in social systems, *emotional deviance* occurs (Thoits, 1990). In non-normative (role) transitions (family exiter), individuals may experience emotional deviance (Thoits, 1990), which can disrupt social systems. Thoits (2004) paves theoretical paths of emotional deviance explored in family exiters’ role transition in this study—labeling by others, labeling of self, seeking validation, and pursuing social change.

## Theoretical models

**Figure 1:**
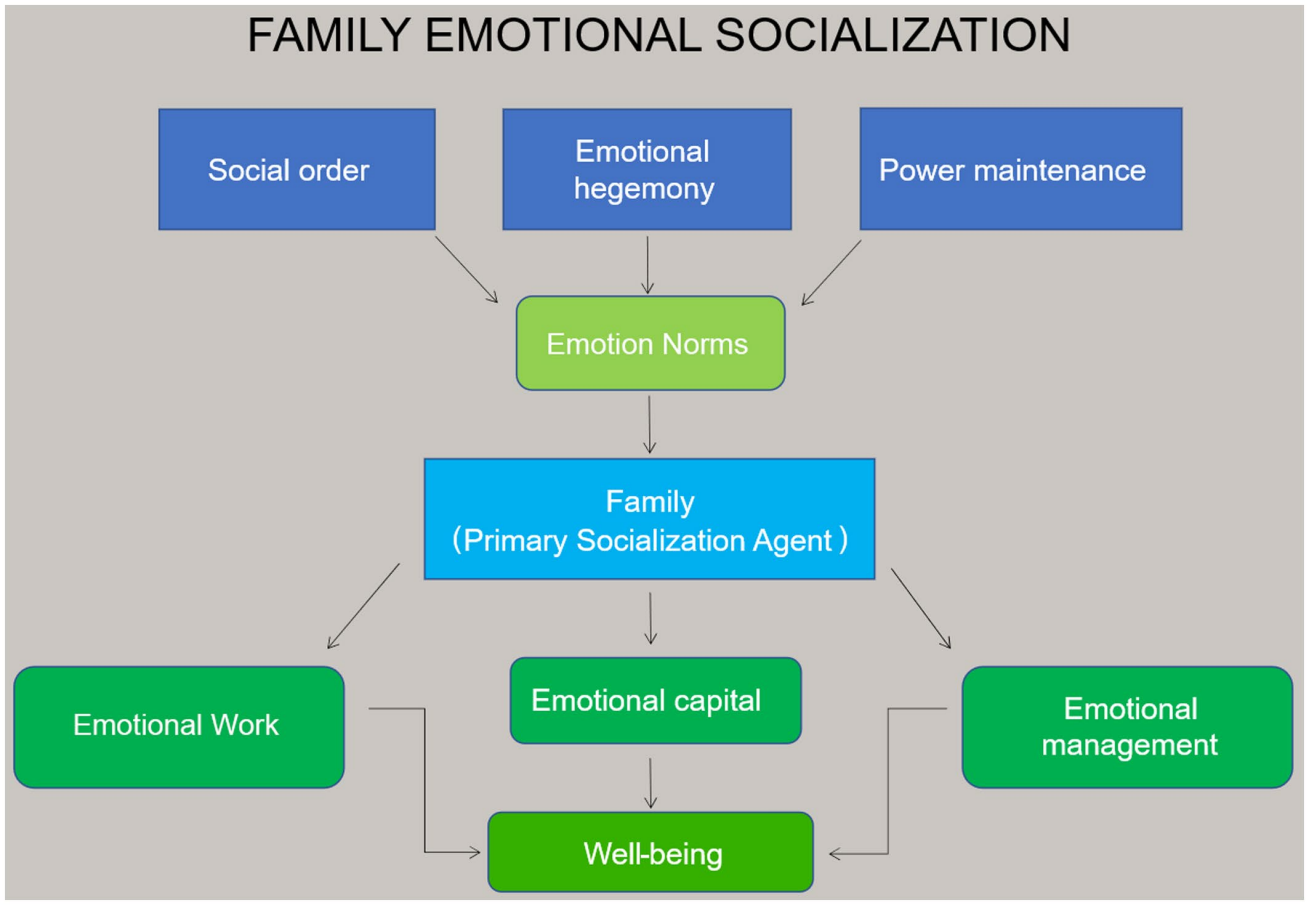
Family emotional socialization.

**Figure 2:**
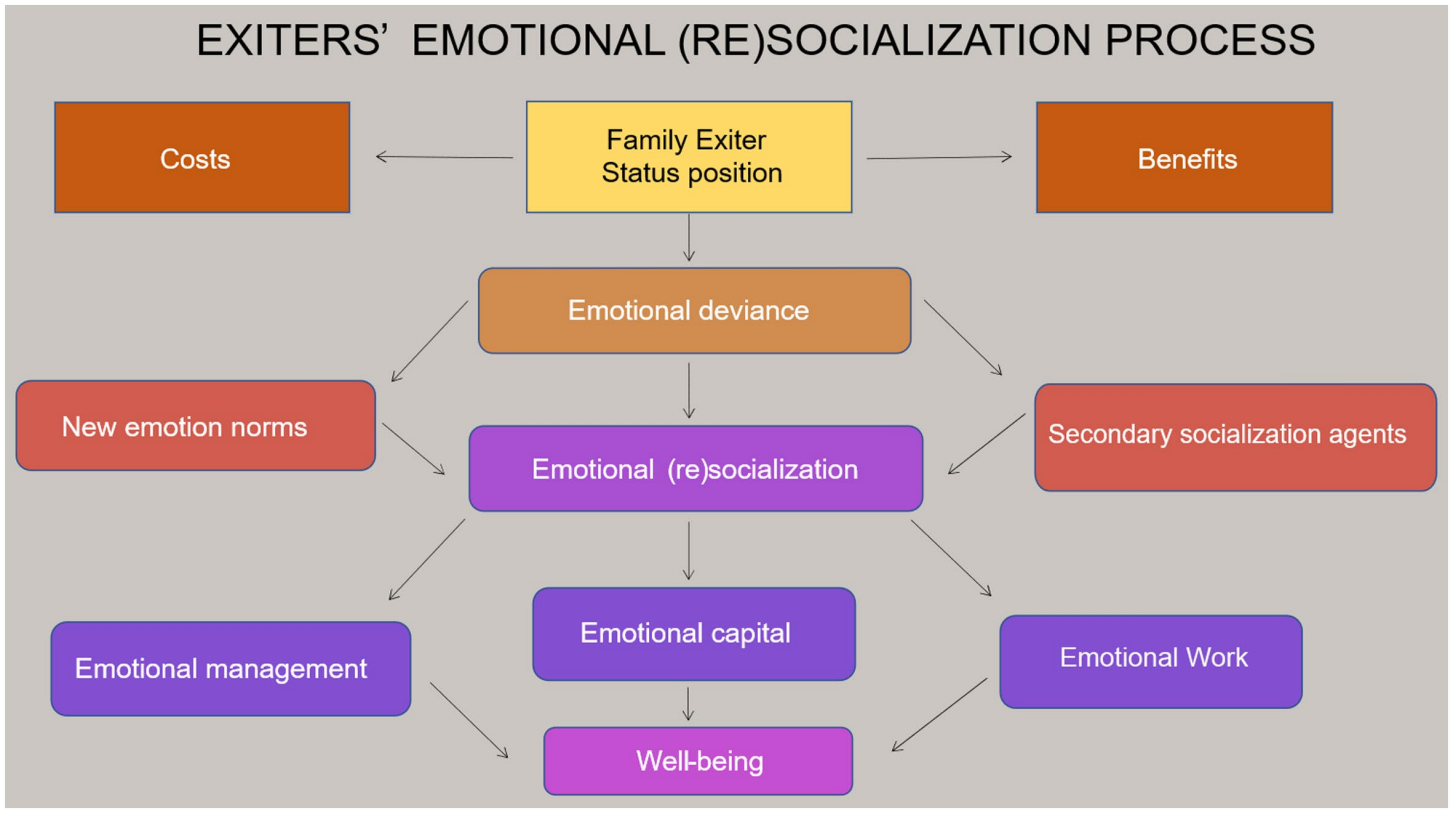
Exiters’ emotional (re)socialization.

To advance theoretical dimensions of these emotion concepts in family exiting, two exploratory models were developed (see Figures 1, 2). The models are the preliminary theoretical advancements of the *early family emotional socialization* and *family exiters’ emotional (re)socialization* processes developed from previous estrangement literature, concepts in the sociology of emotion, and informal online observations of estrangement-focused social media groups. Data collection, analysis, and findings further build upon these models to establish an empirical basis for the lived experiences of family exiters.

Figure 1 illustrates that in the early family emotional socialization process there are both macro and micro level functions. The family system is theorized to be governed by social order, emotional hegemony, and power maintenance. The concept of *emotional hegemony* positions emotions as effective at an individual micro level, but only as part of macro sociocultural operations, and assumes authority of dominant groups while discrediting subordinate groups (Jaggar, 1989). However, as in other hegemonic systems, the possibility of resistance is present. Social order, emotional hegemony and power maintenance influence and shape emotion norms across social institutions. The family system is an early socialization agent that co-constructs and utilizes emotion concepts and micro-level processes (Reay, 2000), such as emotional capital, emotional management and work, and emotional deviance linked to wellbeing.

Figure 2 is a theoretical framework for family exiters’ emotional resocialization process. The family exiter is a status position associated with benefits and costs in the social process of exiting. Initially, exiters will experience emotional deviance (e.g., a transition from family emotion norms and roles) by distancing from the family member(s), and this process continues in varied ways through resocialization. In their non-normative role transition to exiter (Thoits, 1990, 2004), individuals undergo an emotional (re)socialization comprised of constructing new emotion norms and secondary socialization agents (e.g., new support networks). This contributes to the potential accumulation of new emotional capital resources and emotional management and work techniques, which impact wellbeing outcomes.

## Methods

This qualitative research consists of 30 semi-structured online interviews with 4 parents and 26 adult children (non-matched). Participants were purposively sampled from social media groups and organizations, most of which were related to providing support for adult children and parent exiters. A call for interviews was posted to these online groups and/or shared with site and organizational managers to distribute to members. Estranged adult children and parents were always in separate groups. Some other participants responded to a call for interviews on the researcher’s personal social media, as well as sourced from personal growth-oriented groups.

The groups and organizations were selected based on their purpose or intention to discuss issues, relate to others, and support individuals who have experienced estrangement or an exit in an adult child–parent relationship. Given the upward trend in family exiting, there are many social media groups on *Facebook* and a handful of national and international organizations dedicated to this purpose. The criteria in selecting these groups and organizations primarily consisted of their relevant intentional purpose, as well as their availability and willingness to participate in the research. The *personal development* social media groups were also a viable site given they focused on recovery and thriving after trauma-based experiences, whereby some of those experiences were related to family exits.

Due to gateway barriers, a positionality statement was often requested (e.g., state whether the researcher is a family exiter). While this may have influenced the data collection of *parent participants* in this study, this statement was provided upon request. By including a reflexivity statement, researchers make their own approach and stake in the study transparent (Dickie, 2003). This research received Institutional Review Board (IRB) approval from *Anonymous.*

### Participants

Thirty participants (*n* = 30) were interviewed for this study. The majority of participants resided in the United States (87%), followed by the United Kingdom (6.5%) and Canada (6.5%). For detailed sociodemographic data of participants’ gender, sexual orientation, race/ethnicity, education, and age range groupings, refer to Table 1. The age range consisted of 28–77 (*M* = 46.1, *SD* = 11.94). The (sub)sample consisted of *adult children exiters* (*n* = 26), where 30% disconnected from their mother, 23.3% from their father, and 33.3% from both their mother and father. Regarding *parent exiters* (all identified mothers, *n* = 4), 3 were disconnected from their daughters and 1 from their son. Respondents were disconnected for 1–5 years (43.3%), 6–9 years (26.7%), or 10 + years (30%) (see Table 2).

**Table 1:** Participant sociodemographics.

Gender	
Non-binary	1
Male	5
Female	23
Trans Male	1
Sexual orientation	
Bisexual	5
Gay	1
Heterosexual	22
Pansexual	1
Other	1
Race/Ethnicity	
Black	3
Latino	3
White	22
White/British	2
Education	
High School	3
Some college	7
Some graduate school	2
Bachelor’s degree	9
Juris Doctor (JD)	2
Master’s degree	5
PhD/Doctoral degree	1
Not stated	1
Age range	
28–35	6
36–45	12
46–55	5
56–77	7

**Table 2:** Disconnection information.

Disconnected from	
Mother	9
Father	7
Daughter	3
Parents	10
Son	1
Years of disconnection	
1–5 Years	13
6–9 Years	8
10 + Years	9

### Data collection

The following criteria were used to screen participants: (1) at least 18 years of age; and (2) exited at least one parent or adult child for at least 1 year with minimal to no contact. Email contact was initiated by interested participants to ensure they met eligibility requirements. Thereafter, a telephone screening process was conducted to collect sociodemographic and basic information about interested participants’ disconnection process (e.g., duration and who disconnected from). An informed consent was obtained from all interview participants prior to their online interview. All participants’ identities remain anonymous, and pseudonyms are used.

Interviews were conducted online via Zoom, and recordings were professionally and confidentially transcribed. The participants had the option to have their video on or off and were asked their preference before the interview. Online methods enhance understanding of marginalized and underrepresented perspectives from family experiences, as traditional methods do not always afford accessibility to diverse populations (Goldberg and Allen, 2024). Each interview was about 60 min, in addition to a short, unrecorded debriefing. The interviews took place from May to July 2023. The interview questions (see Appendix A) served as a guide for the open-ended, semi-structured interviews. The fundamental ethics in traditional research apply to online interviewing, including (online) consent and data security and storage (Lobe et al., 2020).

### Data analysis

Reflexive thematic analysis (RTA) was used to detect patterns of meaning across the data and identify themes of the processes under examination. Specifically, the six steps of reflexive thematic analysis were followed (Braun and Clarke, 2022): (1) familiarization with the data and memoing; (2) coding the data; (3) generating initial themes; (4) developing and reviewing themes; (5) defining, naming, interpreting themes; and (6) producing the final report. In RTA, *inductive* and *deductive* approaches (used in this study) are part of an interpretative reflexive process, themes are the outcome of coding and iterative theme development, and an emphasis on the importance of researcher subjectivity as an analytic resource (Braun and Clarke, 2021). A research assistant helped with the coding of the data and was trained in the data analysis process, which emphasized analyst triangulation, joint coding, consistent debriefings, and a clear audit trail to establish credibility and dependability (Raskind et al., 2019; Stevano and Deane, 2017). The methods software, *Dedoose*, was used in coding and analysis.

## Findings

The empirical findings were organized by the exiting process across three stages and the *emotion-based themes* at each stage. These processes are segmented into three categories: emotional socialization (early family life), transitional (during the exit), and emotional resocialization (post-exit). The last section provides an overview of *perceived* wellbeing outcomes linked to the second research question in this study. An overview of the empirical findings is provided (see Table 3). The selected quotes are representative of this study’s sample and are grounded in the main themes that emerged from data analysis.

**Table 3:** Overview of findings.

Emotion-based processes	Parents	Adult children	Data interpretation & models
Early emotional socialization	Warmth and closeness Parental conflict/divorce	Abandonment Emotional & Physical Abuse Anxiety Early trauma processing Chaos/volatility Conditional acceptance Gaslighting Warmth and closeness	*Emotion norms* are revealed from early family life. *Emotional capital* is diminished for adult children (forms of abuse). *Power maintenance/relations* in the family are highlighted.
Transitional	Confusion Abandonment Anxiety Chaos/volatility Boundary management Hope/reconciliation	Confusion Anger Anxiety Chaos/volatility Gaslighting Trauma impact Financial abuse Boundary management Hope/reconciliation	*Emotional work* and *management* are realized/initiated. For adult children, trauma impact from early life. For parents, trauma impact of not understanding reason(s) for the exit. *Emotional capital* diminishes for parent exiters.
Emotional (re)socialization	Anxiety Emptiness Emotional Conflict Acceptance Emotional freedom Emotional safety Improved boundaries Grandparent status loss	Anger Anxiety Trauma processing Emptiness Emotional Conflict Lack of emotional ownership Emotional freedom Emotional safety Improved self-worth, self-esteem, personal agency Improved boundaries Acceptance Loss of extended family & siblings Family history loss	Emotion norms from early life are processed to create *new emotion norms* for adult children. *Emotional management* and *work* techniques were further implemented in establishing emotional freedom and safety. *Emotional capital* gained (self-worth, agency, self-esteem, boundary management). *Status position of exiter* (*costs*): loss of extended family, siblings, grandparent role, family history.
Wellbeing outcomes	Social support networks Therapy/Healing Modalities Mental health labeling Stigma (perceived) Peace/relief	Social support networks Therapy/Healing Modalities Mental health labeling Stigma (actual and perceived) Peace/relief	*Secondary socialization agents* are cultivated and formed. *Emotional deviance*: validating exiter role through new partnerships/communities; stigmatizing experiences; mental health labeling. *Positive wellbeing* (all experienced peace/relief); *exiter status position (benefits)*. *Negative wellbeing*: extended emotional management & work to heal; ongoing status costs.

### Early emotional socialization

In considering their early family life, parent exiters emphasize a memory of *warmth* and *closeness* with the child, in addition to conflicts with the other parent. A mother exiter speaks to the *emotional closeness* with her young child while enduring abuse from the child’s father.

“We were extremely close with her [child] growing up. Or I was extremely close. My first marriage to her father, her father was abusive. And she and I were very close. She and I left when she started her senior year in high school. And she and I had always been close.” *Karen, mother disconnected from daughter for 10+ years.*

Another mother exiter recalls her son having high anxiety as a young child, which may have been influenced by divorcing her son’s father early on, and possibly led to the eventual estrangement from her son:

“I think he was struggling as a young child. When I made the decision to leave his dad… I’m going to say we were probably divorced when he [son] was four. Maybe it’s, or five, and maybe the process started between three and four. Which is a pretty formative group of years for a little person. I thought that by divorcing his dad, [it] would help him. I thought it would give him a better life. I do not know if I would change that decision. Maybe I would have had more support. I thought all we had to do was get away from this person [ex-husband], and we would be fine. That wasn’t the case. That person was always going to be in his life…Maybe hindsight is 20/20.” *Monica, mother disconnected from son for 6–9 years.*

The parent exiters (with the exception of one in this study) tend to focus on their separation or divorce from the other parent and their interpersonal conflicts. Overall, the parent exiters expressed confusion as to why their adult child initiated an exit from the relationship.

The adult children in this study expressed a nuanced experience of their *early emotional socialization*. They had all encountered some form of abuse, typically emotional, in early family life. Some highlighted an awareness that *emotional abuse* would not have been considered ‘abuse’ in previous generations. Most of the adult children occupied the generations of Millennial and Generation X, contributing to new understandings of *emotion norms* (including abuse) across generations. *Emotional abuse* generally consisted of neglect, feelings of abandonment, emotional distance, verbal attacks (typically targeting the self-esteem and self-worth of the child), exhibiting emotional authority over the child, and gaslighting.

“It’s a lot of attachment and developmental trauma, emotional abuse, and stuff that I experienced for my entire childhood routinely, like multiple times a day, often there were fucking traumatic things happening.” *Justin, son disconnected from mother for 1–5 years*.

A few adult children also experienced forms of physical abuse linked to emotional abuse.

“…everything started when I was [in] third, fourth grade… emotional abuse started… then physical [abuse] followed… She [mother] would beat me quite a bit. Emotional abuse, manipulation, that all continued throughout elementary school, into middle school and high school. Physical [abuse] stopped right around the time I entered high school. I think it’s because there were two instances where I did not attack her back, but I broke a door out of frustration. The glass broke, and after that and one more incident, she had stopped physically abusing me… and then I also moved out within a year after that to the US overseas… emotional abuse, manipulation, that just continued. As far as I can tell, it continues with my sister.” *Theresa, daughter disconnected from mother for 1–5 years*.

Compounded with the abuse, adult children felt their early family life was clouded by *emotional chaos* and *volatility*, contributing to feelings of *anxiety* and *trauma impact*. Many also felt a *conditional acceptance*—that is, their parents were more concerned about how their child influenced their public role and image, rather than authentically investing in the parent–child relationship. In this way, *emotional capital* was diminished in early life for adult child exiters. Despite their negative experiences, adult children also remembered *warmth* and c*loseness*.

“Growing up, I was my dad’s princess. My dad loved me.” *Elaine, daughter disconnected from father 10+ years.*

Another adult child exiter shares a complex picture of the warmth and closeness:

“The first thing that comes to mind, early on, was some warmth. And then it was sadness and then it was despair, it was indifference, it was the sense of being left out, of not being allowed to grow.” *Kennedy, son disconnected from parents for 1–5 years.*

The emotion-based experiences among adult children and parent exiters in early emotional socialization show some distinct differences, which inform the transitional and post-exiting processes. Parent exiters remember and emphasize positive emotional closeness and parental interpersonal conflicts, while adult child exiters recall an emotional experience that included chaos, volatility, and trauma from the abuse encountered in early life. That said, participants generally recount mixed feelings of warmth and closeness alongside trauma in early life, which informs the emotional complexities of the family exiting process; not as binary emotional experiences but, at different stages, emotions that are in conflicted tension.

### Transitional emotional process

In the transitional process, parents felt a sense of *abandonment* and *confusion*, given that in all the parent exiter cases, they expressed that their adult child *initially* chose to exit the relationship. During this stage, parent exiters’ process was filled with *emotional chaos*, *volatility*, and *anxiety*, mainly in attempting to understand why their adult child decided to disconnect. Parents hoped for reconciliation and made communication attempts, yet they reported their adult child remained generally nonresponsive.

“…I went into depression… What happened? Am I that bad of a parent? I must be that bad of a parent. What did I do wrong? Why did my child do this to me? And I had just remarried… I thought part of that she [daughter] might have hated… But I’m glad that I had because I could not get out of bed. It was very traumatic …she [daughter] said, ‘I just need time.’ I said, ‘That’s fine, but I really would like to talk so we can clear things up.’ She had no interest in that.” *Karen, mother disconnected from daughter for 10+ years.*

For adult children exiters at this stage, *trauma impact* was further realized, leading to feelings of *anger* over their early family life experiences, as well as in recognizing the intensive *emotional work* necessary to heal from the trauma. Conversely, their anger also served as a way to strengthen *boundaries* with their parents. Adult children reported that their parents would contact them during the transitional stage of the exit and violate repeatedly stated boundaries.

“I would say the first few years were pretty hard, and I would think about him [father] a lot, and he would still not respect my boundaries. He would send me a lot of gifts or send my daughter a lot of gifts, and I would have to return them. Sometimes, I’d message him like, ‘Do not send me gifts. We talked about this. If you do not want to have a real conversation with me, you are not allowed to send us gifts.’ That was boundary-setting… There was a lot of having to push back and maintain that wall.” *Sandra, daughter disconnected from father for 10+ years.*

“Even when I went no contact, my mother was pushing the boundaries. It took me moving to a different state, a two-and-a-half-hour drive away for that distance, for that ability to be able to heal.” *Danielle, daughter disconnected from mother for 6–9 years.*

Some adult child participants experienced *economic cut-off*, due to maintaining stricter boundaries with their parents.

“My father provided a lot of financial support to me throughout my life because he is very financially established. And that’s something I’m losing. I do see that very clearly—that I’m losing that access.” *Belinda, daughter disconnected from father for 1–5 years.*

“…she [mother] would provide a lot of finances…when I started school… she would ration it and only send a portion at a time that I needed exactly for school. I could not get financial aid at first, because I’m not a citizen. She said she would help me with it, but she would send me the exact amount… two or three days before a payment was due… and if there was a fight she would not send it… So, I missed a school payment… She’s the one that was deciding how much, how often, or what we’d do with the money.” *Theresa, daughter disconnected from mother for 1–5 years.*

“One negative of it was I was afraid of finances. I’m not quite in poverty, but I’m not much above that. I’m even unemployed now. Those worries are a little amplified when you do not have that family support system. Especially if you are struggling financially, it can be a little worse for you. It can make the stress a little worse. You really have to rely on yourself. But it’s still worth it [exiting].” *Kevin, son disconnected from parents for 6–9 years.*

Parent exiters also made decisions to terminate providing financial support to their adult children when boundaries were violated.

“…when the final disconnection came…I would say, more or less near the eviction… we cut her off financially for lack of payment and cleanliness… evicted her and her boyfriend… I did experience extreme periods where I would think about her continuously. Like I would wonder what she is doing, is she taking care of herself, is she in compromising positions because she does not want to come home…. Then I would think maybe I should call her, or maybe I should not. Or she really needs to learn this time, so I’ll wait for her to call me…” *Kim, mother disconnected from daughter for 1–5 years.*

Adult children learned how to establish boundaries that protected them in their emotional healing, contributing to stronger *emotional management*. Parent exiters continued to feel confusion and anxiety as to why their adult child wanted the distance and attempted to gain understanding. Some adult children and parent exiters reported financial cut-off, pointing to the emotional distancing of the relationship. Adult children sometimes felt a desire for reconciliation yet expressed increased acceptance of “who their parent(s) are,” implying they did not believe their parents would change. While parent exiters did not understand why their adult child initially chose to exit, they also began to accept their own decision to disconnect.

### Emotional (re)socialization

In the post-exit stage, parents and adult children reported an *acceptance* of the relationship exit. Some adult children explained how they felt *compassion* related to the emotion norms that existed in reinforcing their parents’ poor behavior in early family life. That said, adult child exiters did not believe these emotion norms excused poor parenting behavior; therefore, “forgiveness” could not be extended. The concept of *forgiveness* was situated in the idea of offering emotional cleansing to their parent(s). Adult children refused, given their parents’ refusal to take responsibility for their behavior and change.

“To be able to live happily and to heal, you do not have to forgive your parents or connect with them. You can have compassion for them. But there does not need to be forgiveness.” *Veronica, daughter disconnected from parents for 1–5 years.*

The adult children were still processing the impact of their early trauma and shared how emotionally normative it was for their parents to neglect and abuse their children. Additionally, others discussed social awareness of systemic family emotion norms.

“I had no idea I was being abused because my entire contact was my extended family as a child, and everybody in my extended family was doing the same thing to their children. So, I thought that this was normal. It wasn’t until I was a little older, and I was going to my friends’ houses, where I saw, ‘Hey, wait, maybe this is not normal. This is how other people are living.’” *Danielle, daughter disconnected from mother for 6–9 years.*

“Maybe I always knew this, but it just comes to mind how strange, weird, and cruelly we treat children in this country… even that minor awkwardness when someone feels awkward when you kind of share, ‘I do not have a relationship with my parents. I cut them off’… You feel like you are being judged…I really think we treat kids in this society like crap. But it seems very normal for parents to view children not as separate entities but as their own appendage. To challenge or speak out against that, things are going to get awkward in the social circle.” *Kevin, son disconnected from parents for 6–9 years.*

The *emotional conflict* that persisted at this stage was grounded in adult children’s experience of their parents refusing to take responsibility for their behavior. When they attempted to share their early experiences with their parents, their parents dismissed, refused to accept, or avoided the topic altogether. This further cemented adult children’s acceptance of disconnection.

“I would say, ‘What happened when you did not come get me [from school]?’ He [father] would be like, ‘I do not know. I do not remember that.’ There was never any information…he could not talk about anything real, basically.” *Sandra, daughter disconnected from father for 10+ years.*

*Emotional freedom,* achieved through stronger boundary management, contributed to the acceptance of full exit for both parents and adult children. Emotional freedom is implicated by the eventual removal of emotional conflict between adult children and their parents and grants participants a sense of emotional safety. For adult children, it opened up the ability to increase their *self-esteem* and *self-worth*, which was diminished in early family life, as well as the motivation to strengthen *agency* through personal growth endeavors.

“…I see the progress that I’m making and how I’m growing as a human being in just months. I feel so much better about myself, and my self-esteem is improving. I feel moments of joy because I know that I’m not tethered to them [parents]… I do not miss them that much. I really do not. I do not miss talking to them. I do not miss having to do all these mental gymnastics to just get through ten minutes of a conversation…that part I do not miss at all.” *Emily, daughter disconnected from parents for 1–5 years.*

Emotional management also extended to reflecting on their own parenting behavior.

“I feel more in control of my life, I feel safer, I feel I’m able to be a better parent… I view it as I do not want to do the same things that they did, I want to do better, which was kind of the opposite of what I grew up with which was, ‘Well, my parents did this to me, so this is how it is. You guys have it good because it’s not quite as bad,’ versus I’m like, ‘No, I do not want to be anywhere near like that…I do not want to put that on the kids.’ Being able to kind of break that generational trickle-down of choices…” *Kyle, disconnected from parents for 1–5 years.*

Some parent exiters speak to an active choice in being “happy” and the management of feelings in the disconnection process.

“I have to choose to be happy. Because I’m missing a part of my life because she [daughter] and I were so close. I have to decide that I want to be happy. It’s a choice that I have to make. And sometimes it’s every day I have to make that choice. Sometimes as it’s gone on longer…sometimes I do not think about it. It’s easier. But there are times that it’s a decision that I have to make. That I really want to be happy.” *Karen, mother disconnected from daughter for 10+ years.*

Emotional management and work also included the management of feelings of *emptiness* and *loss* connected to *diminished social status* for adult children and parents. For parents, it was the status loss of the grandparent role. For adult children, it was the loss of access to family history and extended family (i.e., aunts/uncles, cousins, siblings, grandparents).

“The main disadvantage would be losing bits of information that would be helpful… Information about family health… With my mom dying when I was 16 and then my grandma also dying, there was a lot of information that died with them. I feel like I’m also on the autism spectrum, and I feel like my dad and my brother are. So, things that he [father] would potentially remember from when I was a kid. Like things about how I was. Or if I ever had chickenpox…” *Denise, daughter disconnected from father for 6–9 years.*

Overall, both adult children and parent exiters expressed the need to let go of the relationship in order to heal and move on, and that required a commitment to emotional management and work techniques, as well as a greater acceptance of the exit.

### Wellbeing outcomes

Adult children and parents exiters found various *social support networks* and *healing modalities* to help with their transition in the exiting process. Most participants sought therapy, spirituality, and/or alternative medicine. Additionally, new networks were cultivated in the form of a new partner, friends, and/or other family exiters through relevant organizations. Parent exiters were less likely to share their exiting experience with others and, thus, had a challenging time finding new social support networks. Some found solace and relatedness participating in family exiting or personal growth organizations, with a few leading within these organizations.

Despite finding new social support networks and therapeutic modalities, exiters often felt stigmatized. Adult children tend to feel both *actual* and *perceived stigma*, likely linked to their willingness to be more open about their exiting experience. Parents, on the other hand, mostly reported feeling *perceived* stigma. They felt they would be stigmatized if they shared their exiter status, therefore, withholding such information in interactions.

“…the disadvantages would definitely be the holidays. It would be functions. People know we have a daughter. And of course they are like, ‘Where’s your daughter?’ I’m like, ‘Oh, she’s around.’ You do not want to say, ‘Well, I have not spoke to her in almost a year’… Another disadvantage is the constant worry. Just because there is estrangement, does not mean you do not worry about her. I worry about her every day… And the stigma. Because when I look at the support group that I joined, even in that particular group, you have people judging each other about, ‘Well, did you try this? Or did you try that? Or maybe if you just reach out to her again for the hundredth time’… There’s a stigma… if there’s estrangement then it must be the mother’s fault.” *Kim, mother disconnected from daughters for 1–5 years.*

While adult children were challenged with the negative impact of actual stigma from others, it also optimized their sense of self-worth in pursuing healing paths and social support networks to feel validation in their experience.

“The rise of your self-worth…it’s really like a rebirth. All the bad things that come with it are just…the bad things that are there, they would not be there if you had this family in your life. I kind of see that for what it is. I have never known until I was like 38, 39, 40 years old and now what self-worth, self-love, and self-esteem truly are…it makes you feel proud of yourself. It makes you feel good.” *Kevin, son disconnected from parents for 6–9 years.*

“Early stage of disconnection, way worse. If I was going to tell anybody, like you got to have your core support system in place before you do this. Without my husband and I being so solid, without having a really solid good therapist and then honestly, without having found the groups, I do not know if I would have done it successfully…” *Maggie, daughter disconnected from father for 1–5 years.*

Some parent exiters were participants and leaders in family exiting organizations where they felt validation and reported elevated wellbeing.

“…this is a group of women who have been through exactly what I have been through… They are at the end. It’s a complete gamut of feelings. That helps each other because I’ve been there. I’ve done that. And the newer women, you just try to be a support to them and tell them that what you are feeling is normal… That was a real support for me. This British woman [leader of organization]… She’s estranged from her own children, and that’s what made her start this group. I really like it. It really helped me.” *Donna, mother disconnected from daughter for 6–9 years.*

“The support that they [organization] are giving the estranged parents… I just cannot say enough. Because I do not think there’s anything like that to help with the recovery for estranged parents… There’s not a lot of support for us because we are isolated. And it’s hard to… How do you tell people? What do you tell people? She’s my only child. I’ve got three stepsons, but they are not mine. I do not have any other children.” *Karen, mother disconnected from daughter for 10+ years.*

Regarding *emotional deviance*, exiters, namely those part of these organizations, reported eventually feeling validated in their exiter role, which increased wellbeing. *Mental health labeling*, among adult children and parents, created tension in the exiting process. When adult children labeled their parents as suffering from a mental illness, mostly undiagnosed and largely *narcissism*, it was linked to not understanding and/or accepting their parent’s behavior in early family life. This provided them with an understanding that further contributed to making sense of their emotional experiences.

“The internet really gave much more…they [people] just thought, ‘Oh, this person was an asshole.’ ‘Or my mom is just a bitch.’ But it’s like, ‘Well, it goes more into that…is this person a narcissist? Does this person have borderline personality disorder?’ Whatever mental health diagnosis. ‘Did they have anybody to support them?’ I know my grandma is also a narcissist, so it’s like my mom did not have the tools to survive my grandma. And then in turn, her trauma turned her into a narcissist.” *Veronica, daughter disconnected from parents for 1–5 years.*

Parents used *mental health labeling* to make sense of why their adult child would exhibit deviant behavior in exiting a parent–child relationship.

“She [daughter] had seen the therapist after I recommended her to her because I had seen that therapist for a long time… My psychiatrist saw her, and my psychiatrist told me that in his opinion ‘this [emotional conflicts] is just going to continue to happen. She’s got borderline personality disorder, and you are going to continue to be hurt. It’s just not going to get better.’” *Donna, mother disconnected from daughter for 6–9 years.*

Emotional deviance also manifested in adult children validating their exiter role by shifting to healthier parenting behavior, different from their parents in early life. Those who decided not to have children felt it emotionally safer to maintain that status, so as to avoid passing down intergenerational trauma.

All exiters in this study reported feeling an overall sense of *peace* and *relief* in their exiting decision. This was linked to the elimination of emotional conflict, healing from early family life trauma, improved boundary management, cultivating new social support, and pursuing personal growth. While this contributed to positive wellbeing, there were ongoing negative impacts. Some of which consist of a prolonged healing process and, at times, contemplating whether they had made the right decision.

“My first year or two were horrific. Horrendous… when she [daughter] had her first baby, I watched him while she went back to work… I smell that baby’s head on my chest to this day, so that was the saddest part to me was not to have my grandson…After the hurt came incredible rage. I still feel anger, but it’s under control. I found a group with a woman in England who has a worldwide meeting of parents of estranged children. That really helped me a lot because it’s very difficult not to understand…you have people that really understand this. It felt like at my age, who knew you would go through this. I’m too old. As far as she’s concerned, I guess, she does not think she’ll see me again until I die. That’s the way it is. She must consider me dead.” *Donna, mother disconnected from daughter for 6–9 years.*

Further, *status loss*—loss of access to grandchildren for parents and extended family loss for adult children—continued to have a negative impact on wellbeing for some exiters. Some adult children expressed how the lack of responsibility continued to weigh on their wellbeing, even though they were moving towards acceptance of never receiving this exchange from their parents.

## Discussion

This research makes worthy contributions to understanding complex emotion-based concepts and processes in family exiting. While there are experiential differences between adult children and parents in this study, there are also common themes in their emotion (re)socialization linked to wellbeing. Early emotional socialization shaped the transitional and (re)socialization stages for both adult children and parents. However, experienced trauma and forms of abuse were more pronounced for the adult children exiters. While previous research highlights abuse as an indicator of estrangement (Agllias, 2015; Blake et al., 2017), this study illuminates the impact of specific variations of emotional abuse (diminished emotional capital) over time, and the emotional work it takes to heal. For parent exiters, age was a factor in the pursuit of healing insofar that the emotional work of healing at a later life stage was challenging to navigate. While past studies point to disparate values in religious, political, and moral ideals as a motivator for estrangement (Davis, 2003; Sucov, 2006), this research did not find that such ideological differences contribute to *primary* reasons for estrangement.

Few studies have investigated intergenerational patterns of estrangement (Becker and Hank, 2021); however, this research contributes to showcasing differences in *emotion norms* across generations and a shift in status-power relations. Adult children acknowledge how forms of emotional abuse masqueraded as acceptable emotion norms in prior generations and have a negative impact on early emotion socialization and (re)socialization. They reported a motivation to avoid reproducing such norms and breaking ‘intergenerational trauma’ to improve their lives and future generations. Parent exiters in this study recognize the emotional limitations of their generation but tend to externalize, rather than internalize the issues, which supports previous research (Carr et al., 2015).

Aligning with previous research (Agllias, 2017; Linden and Sillence, 2021), emotional capital is acquired and compounded for both parents and adult children through new social support networks and partnerships, increased self-worth and self-esteem, pursuit of personal development, healing/therapeutic modalities, and social awareness. Regarding therapy, former research shows that conventional therapy was not always sufficient and can be stigmatizing (Blake et al., 2020); however, this study found that participants who sought therapy largely found it helpful. That said, respondents usually paired it with support organizations and alternative healing modalities. Additionally, new *emotion management* techniques contributed to boundary management and achieving emotional freedom and safety.

Emotional deviance played a processual role in the transitional and (re)socialization stages. In transitioning into a non-normative role of ‘exiter’, most participants experienced stigmatization—labeling of self and labeling from others (Thoits, 2004). Backed by past studies on estrangement (Scharp, 2017), this research also demonstrates how parents and adult children experienced stigma, even if differently. Parent and adult child exiters made sense of their exit by way of *mental health labeling* in an attempt to understand the other’s choices and behavior. In feeling the tension of the exiter role, participants sought support networks that validated their experience and increased social awareness.

Emotion norms in early family life proved to be a disadvantage to adult children and (re)produced emotion-based power struggles between parents and adult children. *Theoretical models* (see Figures 1, 2) in combination with the data contributes to *theoretical findings* along these lines. That is, parents occupy a dominant social role in the family system. Adult children’s exit (emotional deviance) challenges and disrupts the fundamental emotion-structural foundations of family life, thereby propelling parents to safeguard their roles in the hierarchy. Moreover, should parents take responsibility for their parenting behavior and validate their adult child’s feelings and emotional experiences, it potentially would jeopardize their role in the hierarchy and incur emotional consequences.

This study extends theoretical concepts of emotional management and work, emotional capital, and emotional deviance. Unlike most prior work that treats emotional capital as a relatively stable, classed resource (Nowotny, 1981; Reay, 2000), this study demonstrates that it can be disrupted and rebuilt in contexts of family exits. Exiters lost access to inherited emotional capital but actively reconstructed it through new practices of vulnerability, autonomy, and boundary-setting. This adds a dynamic, processual dimension to emotional capital, showing it is not simply transmitted or reproduced, but can be reconstituted in relational fractures.

Building on Thoits (1990, 2004), emotional deviance is often cast as a negative or maladaptive departure from feeling rules, norms, and/or roles. In this study, however, exiters’ “deviant” emotions (e.g., anger, grief, relief) became generative resources for reorienting social belonging and validation. Rather than signaling dysfunction, these emotions were central to emotional resocialization, creating new legitimacy for previously suppressed affect. This challenges the assumption that deviance in emotion norms is purely stigmatizing; instead, it can be an adaptive pathway to emotional agency and identity reconstruction.

This research also extends theories of emotional management and emotional work. Traditionally, Hochschild’s (2012) emotional work framework emphasizes how individuals align their feelings with organizationally or socially sanctioned display rules, often at the cost of authenticity and wellbeing. In family contexts, emotional management typically refers to the regulation of feelings to maintain, sustain, and reinforce familial emotion-based hierarchies. The findings complicate both frameworks by showing that family exiters engaged in counter-emotional management—not to sustain existing norms, but to actively resist and unlearn them. Participants practiced emotional management as a form of boundary work, cultivating authenticity, self-protection, and personal growth.

Likewise, the study refines emotional work theory (Lively and Weed, 2014) by demonstrating that family exiting involves a shift from inauthentic work to agentic work. Rather than exiters performing emotional work to suppress distress or maintain strained family ties, participants engaged in deliberate emotional work to rebuild intimacy skills, negotiate new ties, and recreate emotion norms. By extending emotional management and work, emotional deviance, and emotional capital theories into the context of family exiting, this research demonstrates how these concepts and processes are deeply embedded in family changes and transitions. Further, these concepts are not just about reproducing existing structures but can also become a site of rupture and re-creation, linking directly to the broader process of emotional resocialization.

Alternative family transitions such as empty nest transitions document major changes in familial relationships and are accompanied by complex, mixed emotional outcomes (Hartanto et al., 2024). For instance, empty-nesters may experience loneliness, loss, grief, but also report relief, personal growth, and new forms of wellbeing as they navigate changed family roles (Hartanto et al., 2024). However, this diverges in the mechanisms and broader cultural framing of family exiting, which focuses on the emotional processes and social identity transformation that occur after a stressful exit, detailing unique challenges like stigma, boundary management, and the ongoing negotiation of emotion norms and wellbeing for both parents and adult children. That said, the theoretical contribution of the study’s emotional (re)socialization framework in family exits has potential transferability to alternative family transitions.

### Limitations, future research, and implications

This research has several limitations and strengths. Regarding limitations, the study’s participants are not representative of the broader population (nor intended to be) which limits the generalizability of the empirical findings. Additionally, this study’s sample includes more adult children than parent exiters; therefore, limiting the range of parents’ emotional experiences in family exiting. As well, while this research gathered a diverse sample of exiters across educational background and age, the majority of participants were female, heterosexual, and white. Quantitative studies have examined sociodemographics in this context (Reczek et al., 2022); however, more qualitative research exploring diverse populations and even beyond the U.S., as well as across gender, sexual orientation, race/ethnicity, and class is needed.

More exploration of varied cultural contexts in the family exiting phenomenon would be useful, given that familial emotion norms are influenced by culture. For instance, in Western societies, seeking autonomy, boundary-setting, and personal growth may be more socially promoted compared to non-Western contexts (Mesquita, 2022). Women also appeared to be more active in estrangement groups; thus, identifying methodological approaches to reach more fathers and sons would enhance this body of research. Future research also focusing on the *theoretical expansion* of emotion concepts and processes related to family exiting would add to this timely research topic.

The empirical findings and theoretical models have transferability potential to other groups of exiters, but outcomes may vary depending on resilience and available supports. In particular, the adult children in this study often demonstrated resilience, moving through transitional stage into resocialization, but less resilient exiters may be hindered in earlier phases—in unlearning restrictive norms, establishing new management techniques, or accumulating emotional capital resources. This suggests the importance of expanding research with varied recruitment strategies to capture those whose exits remain incomplete, thereby refining the theory and identifying the conditions that enable or inhibit successful emotional resocialization.

The gateway barriers to these groups were a challenge, which may have been due to the intimate nature and privacy of the family exiting experience. A strength and limitation of this study is the researcher’s positionality. The researcher was often prompted to provide an ‘insider’ statement of whether they were a family exiter. This may have shaped data collection (e.g., less parent exiters); however, it also allowed for greater access to domains restricted to outsiders. Implications of this study point to the development of policies that can support exiters in securing material and emotional resources. The establishment of programs that focus on training mental health professionals in the emotional dynamics of family exits would be advantageous.

## Conclusion

This research contributes to a nuanced understanding of the family exiting process by investigating complex emotion-based concepts and processes, which are at the center of the family system. In unpacking the family exiting process, we can better grasp *how* emotion is constructed and plays a profound role in family systems. Further, the exiting process uncovers particular status-power relations pervasive in family systems and how it is managed, (re)produced, and sustained. By examining the family exiting process, we can more clearly detect the emotional underpinnings, taking the form of normative order qualities, that are accepted, reinforced, and challenged across family generations.

## Data Availability

The raw data supporting the conclusions of this article will be made available by the authors without undue reservation.

## Appendix A: Interview guide

1. Who did you disconnect from?

  a. How long has the disconnection been?

In what forms (e.g., emotional, communicative, physical contact)?

2. What can you tell me about your disconnection process from this person/s?
3. What were your emotional experiences like when you were connected with this family member/s?

  a. Early on, in the same household, and outside the household?

4. What are your emotional experiences like after disconnecting from this family member/s?
5. What, if any, are the disadvantages or consequences of disconnecting from your family member/s?

  a. Did you ever experience stigma from others?

6. What, if any, are the advantages or benefits of disconnecting from your family member/s?
7. What did your support network look like when you were connected to this family member?
8. What does your support network and resources consist of after disconnecting?
9. How had the disconnection process impacted your overall wellbeing?
10. What are your biggest learnings or insights in the disconnection process?

  a. Biggest gains?

11. Finally, what, if anything, do you wish was/had been different in this process for you?
